# Chinese senior primary school students’ civic literacy and its affecting factors

**DOI:** 10.3389/fpsyg.2022.984920

**Published:** 2022-12-15

**Authors:** Yao Yao, Lian-Yu Cai, Mohamed Oubibi

**Affiliations:** ^1^Faculty of Education, Beijing Normal University, Beijing, China; ^2^College of Teacher Education, Education and Human Development, Zhejiang Normal University, Jinhua, China; ^3^Key Laboratory of Intelligent Education Technology and Application of Zhejiang Province, Zhejiang Normal University, Jinhua, China

**Keywords:** senior primary school students, civic literacy, family social economic status, school education, influencing factors

## Abstract

**Introduction:**

Measuring the current situation of civic literacy of senior primary school students (grade 5 and 6) and exploring its affecting factors is the basis for improving the effectiveness of civic education at this stage.

**Methods:**

The research developed the measurement questionnaire through item analysis, exploratory factor analysis, and other methods. It then was distributed nationwide online through stratified and cluster sampling, through which 1642 valid questionnaires were finally collected. The study took mean analysis, difference analysis, correlation analysis, and regression analysis.

**Results:**

The statistical results eventually showed the civic literacy of China’s senior primary school students is, on the whole, at a comparatively high level. Chinese scholars Zhang Jiajun and Xu Jiao combined the particularity of civic literacy to divide it into four dimensions and the performance of the four dimensions of civic literacy is revealed to be: civic affection > civic cognition > civic competence > civic behavior. Further, a significant difference exists in students’ civic literacy by gender (girls’ civic literacy scores *M* = 299.53, *SD* = 28.15, *N* = 785 are significantly higher than the boys’ civic literacy scores *M* = 293.18, *SD* = 32.25, *N* = 857, *t* = 4.263, *p* < 0.01), and whether they have been student leaders (those students who served as student leaders *M* = 304.33, *SD* = 26.57, *N* = 649 those who were not student leaders *M* = 290.86, *SD* = 31.69, *N* = 984, *t* = 9.272, *p* < 0.01).

**Conclusion:**

The family social economic status (SES) and school education were significantly positively correlated with students’ civic literacy. Based on this, primary schools should implement educational compensation for cultivating civic literacy among the socioeconomically disadvantaged senior primary school students and carry out civic education according to the specific conditions of civic literacy of senior students.

## 1. Introduction

Civil society needs citizens with good civic literacy (Gu, 2008). It should be an important objective for school education in china to nurture qualified citizens who can promote social development (Mansilla and Wilson, 2020). China’s civic education development was slow and tortuous as the social and historical conditions restricted it. Until China’s reforms and opening up to the outside world, civic education entered the revival stage. From “Opinions on Improving and Strengthening Political Courses in Secondary Schools” issued by the Ministry of Education in 1980 to “Implementation Outline of Citizen Moral Education” released by the Central Propaganda Department in 2002, then apart from these, “Civic Awareness Education” was incorporated into the strategic themes of the “National Middle and Long-term Education Reform and Development Plan (2010–2020).” These reforms show that civic education’s social atmosphere in China has gradually changed. In 2016, China issued “Key Competencies of Chinese Students,” which included three aspects: cultural basis, independent development and social participation, which manifests synthetically as humanistic connotations, assuming responsibility, and practical innovation, coming to six major literacies. Social participation emphasizes dealing with the relationship between self and society, developing the habits to follow moral codes and conducts of modern civil society, and enhancing the sense of social responsibility (KCRG, 2016).

Contemporary senior primary school students are students growing in a completely new environment of the socialist market economy that is flourishing and developing (Yang et al., 2021). Youngers in his research of new directions for EU civil society support cited that they are important members of civil society and the future of social development in eastern Europe and the western Balkans (Youngs, 2020). After several years of civic education in primary schools, we cannot help asking what the status quo of civic literacy of senior primary school students in China is. To explore such questions in-depth, this study took senior primary school students (Grades five and six) as the research subjects. The core questions of the research include the following:

Question 1: What is the status quo of civic literacy of Chinese senior primary school students?

Question 2: What are the significant factors that affect their civic literacy?

## 2. Literature review

The term “citizen” was bred in the ancient Greek democratic city-states and then developed in modern European countries; that refers to a member of a particular country who has the rights or a person who lives in a particular town or city (Crick, 2007). Other researchers cited that such Citizenship is a status held by a person that entitles the person to certain rights, privileges and protections of a state, and also imposes duties and obligations on the person to the state. A state is a political entity that establishes law, order and provides security to a geopolitical area (Ferrera, 2019; Güney, 2021; Claassen, 2022).

A liberal and dynamic democracy is based on the principles of good governance through vigorous participation of informed citizenry (Neblo et al., 2018). It was not until this term was introduced into China in the late nineteenth century and the early twentieth century. With the continuous development of social and historical culture and the constant integration of civilizations between different countries globally, the connotation and denotation of “citizen” continued to expand. The definition of citizenship has historical and cultural particularity. It becomes even more complex when we consider the different levels of community, super-national (e.g., the European Union), and the world (Tan, 2010). Under the background of globalization, citizenship is in transformation. Audrey Osler believed that status, feeling and practice are the three indispensable and complementary parts of citizenship (Osler and Stucki, 2012). Feng Jianjun proposed that citizens should be the integrity of the Citizens of Rights, the Citizens of the Nation, the Social Citizens, and the World Citizens. The construction of civic education goals should also be multidimensional (Feng, 2011). However, no matter what level of educational goals, civic education must first be based on the general promotion of individual civic literacy.

Literacy traditionally meant the ability to read and write. The modern term’s defined as the knowledge, skills and attitudes that students need to work effectively in a diverse society (Bauerlein, 2012), and it has meaning has been expanded to include the ability to use language, numbers, images, computers, and other basic means to understand, communicate, gain useful knowledge, solve mathematical problems and use the dominant symbol systems of a culture (UNESCO, 2006). Civic literacy in this article refers to literacy related to the connotation of citizenship. The early clear definition of civic literacy in China was found in Cheng Youxin’s study, and he believed that modern civic literacy is a combination of cultural literacy, moral literacy, legal literacy and political literacy with equality as the core; that is multifaceted habits, behaviors, norms and knowledge of different levels, such as cultural level, moral level, legal level, and political level (Cheng, 1996). However, Li Fang proposed comprehending civic literacy from three kinds of social relations: citizens and the state, citizens and society, and citizens and citizens. Specifically, it manifests the good quality, patriotic quality and political participation quality at the level of citizens and state; the social participation quality, independent quality and self-improvement quality at the level of citizens and the society; as well as the honesty quality, amicable quality, cooperation quality and lenient quality at the level of citizens and citizens (Li, 2006). Wang Chunying defined civic literacy from the political point of view, divided into two aspects: citizen consciousness and citizen competence. The former mainly includes the consciousness of rights and responsibility, rule consciousness, subjective self-reliance consciousness, win-win consciousness, and social morality consciousness. The latter mainly includes political cognitive ability, rational communication ability and rational judgment ability (Wang, 2010). In addition, the International Association for the Evaluation of Education Achievement (IEA)‘s civic literacy evaluation framework in the Third International Civic and Citizenship Education Study (ICCS2009) showed that civic literacy is composed of three parts, which include content domains, affective-behavioral domains and cognitive domains, and the content domains are composed of civil society and systems, civic principles, civic participation and civic identities (Schulz et al., 2016). Based on Bloom’s taxonomy of educational objectives, Chinese scholars Zhang Jiajun and Xu Jiao combined the particularity of civic literacy to divide it into four dimensions, namely, civic cognition, civic emotion, civic competence and civic behavior (Zhang and Xu, 2016). Although China and abroad are different in their formulations and definitions of civic literacy’s connotation, they all focus on the relationship between civic rights and obligations and emphasize the connotations of politics, law, morality and participation.

The development of modern democratic society has made civic education the focus theme in the international community and educational development in various countries and regions. However, it should be noted that the civic values and the course contents and implementation of civic education are inevitably different among different countries of the world. Paul Morris pointed out that the formal civic curriculum in China, Chinese Hongkong, Chinese Taiwan and Thailand is usually a series of ethical education related to “good citizens” and “common interests.” At the same time, the United States and Australia focus on studying democratic values, the history of national constitutionalism and civil rights (Paul and John, 2001).

The media literacy of citizens has also attracted the attention of scholars in China, as Cai Lianyu considered that media education research should be expanded from the specialized media studies and vigorously studied and implemented in citizen moral education (Cai, 2007). Chen Xiaohui also believed that media literacy education as a subsystem of civic education should improve civic literacy to improve the citizens’ ability to acquire, select, evaluate, participate, and create media information (Chen et al., 2013).

The academia in China has also paid close attention to the theoretical research on public awareness of citizens and civic literacy, even though there is a lack of systematic research on civic literacy, especially empirical research. With this background, the China National Condition Research Center of Peking University launched a large-scale investigation based on nationwide probability sampling in 2008, namely, the 30th Anniversary of the Reform and Opening Up Policy which was the Annual Survey of Chinese Citizenship Consciousness. This survey aimed to investigate the development status of citizenship consciousness in national identity and pride, civil liberty, democratic consciousness, legal consciousness, rights and obligations of citizens, justice consciousness, citizen participation, and trust and to explore further the impacts and changes on citizenship consciousness brought about by China’s modernization. This survey filled the gap in a systematic empirical study of citizenship consciousness in China (Shen et al., 2009). In addition, Zhao Lingyun conducted a study on Shanghai teenagers based on three aspects of moral literacy, legal literacy and political literacy (Zhao et al., 2011), and Xie Weiguang and Zhao Lingyun conducted a questionnaire survey and interviews with some families in Shanghai to investigate the role of the family in the construction of teenagers’ civic literacy (Xie and Zhao, 2012).

On account of the differences in cultural and ideological traditions among countries globally, it is necessary to make localization design when constructing a civic literacy measurement scale, and it is impossible to have a completely unified one. As Lee Wingon pointed out, East Asian countries and regions influenced greatly by Confucian culture had unique citizenship and civic education characteristics compared to Western countries. They emphasized the harmonious relationship between individuals and others, individuals and society and spirituality (Lee, 2004). The extant representative civic literacy scale in China was developed by Zhang Jiajun and Xu Jiao, the “Civic Literacy Scale of Primary and Secondary Students.” This scale comprises four dimensions: civic cognition, civic emotion, civic competence, and civic behavior. The measurement data have shown good reliability and validity (Zhang and Xu, 2016). Beyond that, China lacks a relatively mature civic literacy scale, especially for specific groups like senior primary school students.

Generally, since the Reform and Open, along with the progressing elaboration of social politics, economy and education system, the research on civic education in China has also presented a prosperity trend, and a large number of translations, monographs, academic papers and other achievements have sprung up during this period. However, the study of civic literacy is slightly weaker than that of civic education. The theoretical research about civic literacy in China is mostly monographic studies focusing on one aspect of civic literacy’s connotation. These include a focus on civic awareness and competence or a single type of civic literacy, like environmental, media, health, and moral literacy. In contrast, the systematic studies on civic literacy are relatively fewer. From the point of view of applying research methods, most of the existing studies belong to theoretical research, and empirical research based on data surveys is relatively deficient. Therefore, this study aimed to investigate the current status quo and affecting factors of senior primary school students’ civic literacy in China, thereby providing data to support the effectiveness of civic education.

## 3. Methodology

### 3.1. Sample information

This research chose China’s senior primary school students (Grade 5 and 6) as study objects, used stratified and cluster sampling methods, and took primary school as the cluster unit. Sampling units covered China’s eastern, central and western areas and involved six province-level administrative regions, including Beijing, Sichuan, Jiangxi, Zhejiang, Shandong and Hubei between September to the end of December 2021. Samples were from the fifth and sixth graders of primary schools in the countryside, townships, counties and cities of the provincial regions above. A total of 2,100 questionnaires were distributed online, all participant in the study offered an informed consent form, and 1,995 were collected. After 353 invalid questionnaires were eliminated because of the missing of many information’s, there were 1,642 valid questionnaires left and it is representative for the general population and the minimum sample size for the representative sample with fraction size 323 (confidence Level = 95%, Margin of Error = 5%, Population Proportion: 50% and population size = 1995), and the effective recovery rate was 78.19%, and the difference of gender 52.19% were Male and 47.81% females does not affect the results of the study and there’s no significant difference in the grade 5 (51.22%) and grade 6 (48.54%). All participants were guaranteed anonymity and their response will be used for academic study. The Ethics Committee of the Zhejiang Normal University’s, College of Teacher Education approved the study and participant consents and the study followed the Declaration of Helsinki.

Demographic information on the questionnaire included gender, origin’s, whether the only child, grade and whether they were student leaders. The specific sample information is shown in Table 1.

**Table 1:** Sample demographic information.

	Category	Samples	Percentage		Category	Samples	Percentage
Gender	Male Female Missing	857 785 0	52.19 47.81 0.00	Grade	5 6 Missing	841 797 4	51.22 48.54 0.24
Home	Countryside Township County City Missing	420 211 280 722 9	25.58 12.85 17.05 43.97 0.55	Whether the only child	Yes No Missing	725 903 14	44.15 54.99 0.86
				Whether Student leaders	Yes No Missing	649 984 9	39.52 59.93 0.55

### 3.2. Development of the questionnaire

The questionnaire was developed by combining self-compilation and adaptation methods. The construction of the scale was based on the four-dimensional structure model of Zhang Jiajun and Xu Jiao, in which civic literacy was split into four first-degree indexes, including civic cognition, civic emotion, civic competence and civic behavior. The scale used the seven points by Likert. Reverse questions were set in all four dimensions. There were 64 items on the initial scale with first 16 questions for civic cognition and participants were asked such questions as (“e.g: A good habit of civility and courtesy is a kind of self-cultivation?” – “e.g: It is our duty to care for public property), 15 items for civic emotion as (“e.g: I think China is a great country?”), 15 items for civic competence as (“e.g: I can actively give ideas, think of ways, and contribute to collective activities?” – “e.g: I can actively express my opinions in class?”) and 18 items for civic behavior as (“e.g: I’m concerned about environmental pollution” – “e.g: I have never intentionally damaged public facilities”) (Zhang and Xu, 2016).

The questionnaire test adopted a cluster random sampling method to select two primary schools in Jinhua city, Zhejiang province, choosing grade 5–6 students as respondents. A total of 210 questionnaires were distributed, and 207 were collected. After eliminating 9 missing data questionnaires, there were 198 valid questionnaires left, and the effective rate was 94.29%. After numbering the valid questionnaires, SPSS22 was used to encode the questionnaires and input data. Then, the research adopted methods of item analysis, exploratory factor analysis and reliability analysis to modify the questionnaire.

#### 3.2.1. Item analysis

Extreme group comparison was used to analyze items. Each dimension was divided into the higher scores group (the first 27%) and the lower scores group (the last 27%). Then, the average scores of each item of the higher and lower scores group were analyzed with Independent-Samples T-Test. If the mean difference is non-significant, the item lacks discrimination. The results showed that the average scores of each item of higher and lower score groups of each dimension had a significant difference, which meant that each item had good discrimination.

#### 3.2.2. Exploratory factor analysis

The construction of the scale was based on the four-factor structure theory of current research. The good structural validity of the theoretical structure model has been confirmed. Therefore, during the exploratory factor analysis, items of each factor could be implemented separately (Qiu, 2009). Before the analysis, KMO and Bartlett tests were conducted on items of each dimension to decide whether the item was suitable for factor analysis. The extraction of factors used the principal component analysis method. The standard decided the number of factors that the eigenvalue is greater than 1. The maximum variance method was adopted to implement factor rotation. The number of free exploration factors could also be observed with the screen plot. In the process of model modification, items whose factor load was less than 0.4 and more than 0.3 on multiple factors were eliminated. These steps above were repeated every time an item was deleted until a relatively stable factor structure had been found. The details of the four first-degree factor dimensions’ exploratory factor analysis are described below.

##### 3.2.2.1. Civic cognition

The test results show that KMO is equal to 0.729, and the degree of sphericity of Bartlett is less than 0.001, which indicates that exploratory factor analysis is feasible. The number of factors whose eigenvalue is greater than 1 is six. The total explained variance is 54.14%. Combined with the observation of the screen plot, three factors were extracted. After several model modifications, five items were deleted in the end from 16 items. The three factors were named “moral cognition,” “cognition of politics and law (PL cognition),” and “public cognition.” The total explained variance is 49.27%.

##### 3.2.2.2. Civic emotion

The test results were that KMO is equal to 0.763, and the degree of sphericity of Bartlett is less than 0.001, which indicates that exploratory factor analysis is feasible. The number of factors whose eigenvalue is greater than 1 is five. The total explained variance is 62.38%. Combined with the observation of the screen plot, three factors were extracted. After several model modifications, four items were deleted in the end from 15 items of civil emotion. The three factors extracted were named “national identity,” “social responsibility,” and “collectivism.” The total explained variance is 57.63%.

##### 3.2.2.3. Civic competence

The test results were that KMO is equal to 0.840, and the degree of sphericity of Bartlett is less than 0.001, which indicates that exploratory factor analysis is feasible. The number of factors whose eigenvalue is greater than 1 is five. The total explained variance is 63.30%. Combined with the observation of the screen plot, four factors were extracted. After several model modifications, two items were deleted in the end from 15 items. The four factors extracted were named “rights protection,” “moral judgment,” “moral choice,” and “cooperation.” The total explained variance is 62.59%.

##### 3.2.2.4. Civic behavior

The test results were that KMO is equal to 0.829, and the degree of sphericity of Bartlett is less than 0.001, which indicates that exploratory factor analysis is feasible. The number of factors whose eigenvalue is greater than 1 is six. The total explained variance is 67.108%. Combined with the observation of the screen plot, three factors were extracted. After several model modifications, five items were deleted from 18 items. The three extracted factors were “public life,” “social participation” and “moral behavior,” respectively. The total explained variance is 62.457%.

After the exploratory factor analysis, the conclusion of the factor structure of the measurement scale of civic literacy of senior primary school students is shown in Table 2.

**Table 2:** The factor structure of the measurement scale of civic literacy of senior primary school.

Dimension	Factor structure	Item (factor load)
Civic cognition	Moral cognition	R14(0.771), R12(0.745), R6(0.715), R15(0.534)
	PL cognition	R1(0.656), R5(0.681), R11(0.490), R2(0.481)
	Public cognition	R9(0.757), R7(0.520), R13(0.578)
Civic emotion	National identity	Q19(0.815), Q18(0.783), Q20(0.774), Q22(0.768), Q24(0.545)
	Social responsibility	Q28(0.770), Q27(0.744), Q29(0.682), Q26(0.539)
	Collectivism	Q23(0.664), Q30(0.657)
Civic competence	Cooperation Moral choice Moral judgment Rights protection	N42(0.788), N40(0.756), N39(0.751), N43(0.735), N38(0.719), N41(0.668) N44(0.716), N34(0.699), N45(0.594) N37(0.832), N35(0.714) N32(0.817), N33(0.677)
Civic behavior	Public life Social participation Moral behavior	X62(0.858), X63(0.791), X61(0.682), X59(0.653) X51(0.782), X49(0.737), X52(0.718), X48(0.654), X64(0.616) X57(0.836), X58(0.790), X60(0.600), X55(0.473)

#### 3.2.3. The formation of the formal questionnaire

Exploratory factor analysis and reliability analysis, the measurement scale of civic literacy of senior primary school students was eventually formed, which contained three-level indexes (Table 2). The formal questionnaire was divided into two parts with 64 items and the final questionnaire consist of 48 items after eliminating 16 items and the questionnaire consist of 11 items for civic cognition, 11 items for civic emotion, and 13 items for civic competence and 13 items for civic behavior giving 48 final items.

### 3.3. The reliability and validity of the questionnaire

The statistical results of the formal investigation showed that the internal consistency reliability of the measurement scale of civic literacy of senior primary school students was (cronbach α = 0.910), the reliability coefficients of civic cognition, civic emotion, civic competency and civic behavior were (cronbach α = 0.633, 0.773, 0.781, and 0.858) respectively, and this showed that the reliability of the whole scale was good. At the same time, experts were consulted for advice on the process of developing the questionnaire. On this basis, the questionnaire was compiled and revised, which assured the expert validity of the whole questionnaire. The factor analysis results in the test phase showed that the questionnaire had good construct validity.

## 4. Results and statistical analysis

The research analyzed the current situation of the civic literacy of senior primary school students in six provinces of China through survey data. It explored the main factors that impact the civic literacy of senior primary school students, using difference analysis, correlation analysis and regression analysis methods.

### 4.1. Literacy status quo

The scale used the seven points by Likert, a total of 48 items, ranging from 48 to 336. The specific points are shown in Table 3.

**Table 3:** The status quo of senior primary school students’ civic literacy in China *N* = 1,642.

Factors	Mean	SD	Number of items	Mean of items	Skewness	Kurtosis
Civic literacy	296.22	30.51	48	6.17	−1.515	3.659
Civic cognition	68.59	6.93	11	6.23	−1.451	3.355
PL cognition	23.28	4.02	4	5.82		
Public cognition	18.51	3.07	3	6.17		
Moral cognition	26.80	2.54	4	6.70		
Civic emotion	69.49	8.14	11	6.32	−1.913	5.488
National identity	32.64	4.20	5	6.53		
Social responsibility	23.97	4.18	4	5.99		
Collectivism	12.88	2.02	2	6.44		
Civic competence	80.24	9.81	13	6.17	−1.447	3.085
Cooperation	36.02	6.29	6	6.00		
Moral choice	18.78	2.96	3	6.26		
Moral judgment	13.41	1.79	2	6.71		
Rights protection	12.03	2.40	2	6.02		
Civic behavior	77.90	12.27	13	5.99	−1.216	1.416
Social participation	28.53	6.27	5	5.71		
Public life	25.20	3.82	4	6.30		
Moral behavior	24.17	4.65	4	6.04		

As shown in Table 3, the overall level of civic literacy of senior primary school students in China is good. A paired-Sample T-test was used to test the average value of the four dimensions of civic literacy, and the results show that the mean value of each dimension had a significant difference of (*p* < 0.01). Comparing the four dimensions of civic literacy and ranking them according to the mean value, the literacy level goes from civic emotion to civic cognition and then down to civic competence and finally to civic behavior.

### 4.2. Difference analysis

#### 4.2.1. Family social-economic status

Family social-economic status, called family SES, is a vital index to influence students’ development. Studies showed that family SES is related to the development of students. In the early studies, the most representative and influential being “the Coleman Report” (Coleman, 1966) and “the Plowden Report” (Peaker, 1971), both pointed out that family background plays a directly explanatory and predictive role in students’ achievements. In measuring family SES in domestic and international studies, parental education, occupation and income are generally used as indicators. This study adopted the general measuring method of family SES and analyzed the difference in civic literacy of senior primary school students in this dimension. Firstly, we calculated the total scores of students’ family SES and found the first and last 27% scores, then divided family SES into high, medium and low levels, and finally used one-way ANOVA of independent samples to test whether there was a significant difference of civic literacy of students among different family SES groups. The test for homogeneity of variance showed that sig = 0.075 > 0.05. The results of one-way ANOVA showed that there was a significant difference in the scores of civic literacy among different family SES groups [*F*(2,1,302) = 44.772, *p* < 0.01]. The method of Tukey was used to check out afterwards, the result of which is in (Table 4) showed that the total points of civic literacy of high-level family SES are significantly higher than the low-level and the medium-level family SES, and the total points of civic literacy of medium-level family SES are significantly higher than that of the low-level family SES.

**Table 4:** Multiple comparisons of civic literacy among different families SES.

Dependent variable: civic literacy level							
	**(I) Family SES level**	**(J) Family SES level**	**Mean difference (I,J)**	**SE**	**Sig.**	**95% confidence interval**	
						**Lower bound**	**Upper bound**
Tukey HSD	Low	Medium	−10.794^*^	1.805	0.000	−15.030	−6.558
		High	−18.999^*^	2.036	0.000	−23.776	−14.222
	Medium	High	−8.205^*^	1.941	0.000	−12.760	−3.650

*The mean difference is significant at the 0.05 level.

#### 4.2.2. School education

Generally, school education is another important factor that influences the development of students. Is there any significant difference in students’ civic literacy in different schools? To find out, the research conducted statistical tests on otherness. We gave a series of index numbers to individual educational items. By summing up all the school education items, we got the variable called school education, then using the same method, we divided the school education level and got three groups: the high, medium and low-level school education groups. Finally, we conducted a univariate dependent sampling analysis of variance for each school education group. Using the data collected, the homogeneity of variance test showed that sig = 0.000 < 0.05, which means variances are uneven among groups. The results of the One-way ANOVA indicated that there were significant differences in civic literacy under different levels of school education (*F* (2,1,630) =137.292, *p* < 0.01), and the results of the robustness examination (Table 5) further support this finding. The research used Dunnett’s T3 method to perform the post-test when the assumption of the homogeneity of variance was violated. The result (Table 6) showed that the total score of civic literacy of the high-level school education group was significantly higher than that of the low-level and the medium-level school education groups. The total score of civic literacy of the medium-level school education group was also significantly higher than the low-level school education group.

**Table 5:** Robust tests of equality of means.

	Statistic^a^	df1	df2	Sig.
Welch	178.229	2	992.860	0.000
Brown-Forsythe	144.983	2	1272.308	0.000

^a^asymptotically F distributed.

**Table 6:** Multiple comparisons of civic literacy among different school education.

Dependent variable: civic literacy level							
	**(I) School education level**	**(I) School education level**	**Mean difference** **(I,J)**	**SE**	**Sig.**	**95% confidence interval**	
						**Lower bound**	**Upper bound**
Dunnett T3	Low	Medium	−13.826^*^	1.851	0.000	−18.253	−9.398
		High	−31.588^*^	1.807	0.000	−35.911	−27.266
	Medium	High	−17.763^*^	1.385	0.000	−21.075	−14.450

* The mean difference is significant at the 0.05 level.

#### 4.2.3. Multiple comparisons of civic literacy

The differences in individual natural attributes (gender, origin, grade, etc.) were also analyzed. In the independent sample T-test regarding gender, girls were set to “1,” and the boys were set to “2.” The data analysis showed that the girls’ civic literacy scores (*M* = 299.53, SD = 28.15, *N* = 785) are significantly higher than the boys’ civic literacy scores (*M* = 293.18, SD = 32.25, *N* = 857). The differences in the average scores between the two groups were 6.36, *t*(1640) = 4.263, *p* < 0.01. Using the same test method, the statistical results showed that the civic literacy scores of those students who served as student leaders (*M* = 304.33, SD = 26.57, *N* = 649) were significantly higher than those who were not student leaders (M = 290.86, SD = 31.69, N = 984). The differences in the average scores between the two groups were 13.46, *t*(1631) = 9.272, *p* < 0.01. Based on the finding of univariate dependent sampling analysis of variance in terms of origins, students’ civic literacy does have significant differences because of different origins [*F*(3,1,629) =3.303, *p* < 0.05]. The research used the method of Tukey to perform post-tests. The result (Table 7) showed that city and county students’ civic literacy scores are significantly higher than countryside students. The civic literacy scores of county students are significantly higher than those of township students, and no significant difference between other groups. Furthermore, statistical results showed no significant difference in the civic literacy scores between an only child and a non-only child and between grades five and six.

**Table 7:** Multiple comparisons of civic literacy among different origins location.

Dependent variable: civic literacy level							
	**(I) origins**	**(J) origins**	**Mean Difference (I,J)**	**SE**	**Sig.**	**95% confidence interval**	
						**Lower bound**	**Upper bound**
Tukey HSD	Countryside	Township	0.058	3.549	0.987	−6.904	7.020
		County	−7.401^*^	3.064	0.016	−13.410	−1.391
		City	−5.412^*^	2.248	0.016	−9.821	−1.003
	Township	County	−7.459^*^	3.679	0.043	−14.675	−0.243
		City	−5.470	3.033	0.072	−11.420	0.479
	County	City	1.988	2.448	0.417	−2.813	6.790

* The mean difference is significant at the 0.05 level.

### 4.3. Correlation analysis

Based on the analysis of the differences, this study investigated the correlation between the civic literacy of senior primary school students and their family SES. To begin with, we analyzed the correlation between different family SES and different levels of civic literacy. In the same way, this study divided the students’ civic literacy into three levels: high, medium and low, and then made a cross-tabulation of family SES and civic literacy (Table 8). As can be seen from the cross-tabulation, the low civic literacy group takes the high proportion of the low family SES group, the medium civic literacy group takes the high proportion of the medium family SES group, and the high civic literacy group takes the high proportion of high family SES group. From the overall data distribution, it can be inferred that there is certain relevance between the civic literacy of senior primary school students and their family SES.

**Table 8:** Family SES level * Civic literacy level cross-tabulation.

			Civic literacy level		
			Low	Medium	High
Family SES level	Low	Count	156	209	64
		% within family SES level	36.4%	48.7%	14.9%
	Medium	Count	127	267	147
		% within family SES level	23.5%	49.4%	27.2%
	High	Count	52	128	155
		% within family SES level	15.5%	38.2%	46.3%

The chi-square independent test between different family SES groups and different levels of civic literacy was conducted to examine whether there was a significant correlation between the two grouping variables. The results showed that χ^2^(4, *N* = 1,305) = 106.106, *p*<0.001, Cramer’s V = 0.202. Since the two variables belong to ordered variables, we can also use the Spearman rank correlation to show the degree of relevance between the two, and the data showed that ρ = 0.271, *p* < 0.001. The results demonstrated a significant correlation between different family SES groups and different levels of civic literacy.

Finally, the study further analyzed the interrelationship between the civic literacy of senior primary school students and their family SES, and the results showed (Table 9) that the Pearson correlation coefficient of family SES and total civic accomplishment is r(1303) = 0.254, *p* < 0.001. According to the general interpretation of the correlation coefficient, when the coefficient is lower than 0.2 or 0.3, it indicates a low correlation. When the coefficient is between 0.3 to 0.6, it indicates a moderate correlation; when the coefficient is between 0.6 and 0.8, it indicates a strong correlation; when the coefficient is above 0.8, it indicates a high strength correlation (Peter, 2009), and the two have a significantly positive low correlation. According to Cohen’s rule of thumb, the r values of the small, medium, and large effects are ±0.10, ± 0.30, ± 0.50 (Chen, 2015), this correlation is close to moderate effect size.

**Table 9:** Correlation between family SES and civic literacy.

	Civic literacy	
Family SES	Pearson correlation	0.254^**^
	Sig. (two-tailed)	0.000
	N	1,305

**Correlation is significant at the 0.01 level (two-tailed).

In addition, we conducted an in-depth correlation analysis of the three indexes of family SES (parental education, parental occupation and household income) and the four dimensions of civic literacy. The statistical results (Table 10) show that these three indexes have a significantly positive low correlation with civic competence and civic behavior. In contrast, they have a weak correlation with civic cognition. Parental education and parental occupation have non-significant associations with civic emotion, and the correlation between civic emotion and household income is rather weak. By comparing these three indicators and civic competence and civic behavior, we found that parental education has the strongest correlation with civic competence and civic behavior, followed by parental occupation, then household income.

**Table 10:** Correlation between family SES indicators and civic literacy dimensions (Pearson).

	Parental education	Parental occupation	Household income	Civic cognition	Civic emotion	Civic competence	Civic behavior
Parental education	1	–	–	–	–	–	–
Parental occupation	0.698^**^	1	–	–	–	–	–
Household income	0.393^**^	0.407^**^	1	–	–	–	–
Civic cognition	0.104^**^	0.099^**^	0.088^**^	1	–	–	–
Civic emotion	−0.006	−0.019	0.068^**^	0.539^**^	1	–	–
Civic competence	0.225^**^	0.199^**^	0.179^**^	0.494^**^	0.568^**^	1	–
Civic behavior	0.285^**^	0.251^**^	0.225^**^	0.450^**^	0.510^**^	0.716^**^	1

**Correlation is significant at the 0.01 level (two-tailed).

The study also conducted a correlation study between school education and senior primary school students’ civic literacy. Following the same steps, firstly analyzed the correlation between school education and civic literacy and more details of the cross-tabulation in (Table 11). From the overall data distribution of the cross-section, we could see there was a certain relevance between school education and civic literacy. Secondly, a chi-square independence test was conducted among different school education groups and civic literacy groups. Results showed that χ^2^(4, *N* = 1,633) = 246.355, *p* < 0.001, Cramer’s V = 0.275. We used the Spearman rank correlation to show the degree of relevance between the two, and the data showed that ρ = 0.362, *p* < 0.001. The results indicated a significant correlation between different levels of school education groups and different civic literacy groups. Thirdly, the study correlated school education and senior primary school students’ civic literacy. Results in the table (Table 12) showed that the Pearson correlation coefficients of the school education and civic literacy were r(1633) = 0.382, *p* < 0.001, both are significantly positive and moderately correlated with moderate effect size. At the same time, we conducted an in-depth correlation analysis of the four dimensions of civic literacy and school education. From the statistical results (Table 12), we could deduce significant positive correlations between the two dimensions of civic literacy and school education.

**Table 11:** School education level* Civic literacy level cross tabulation.

			Civic literacy level		
			Low	Medium	High
School education level	Low	Count	210	219	67
		% within school education level	42.3%	44.2%	13.5%
	Medium	Count	201	379	165
		% within school education level	27.0%	50.9%	22.1%
	High	Count	30	153	209
		% within school education level	7.7%	39.0%	53.3%

**Table 12:** Correlation between school education and civic literacy and its dimensions (Pearson).

	School education	Civic literacy	Civic cognition	Civic emotion	Civic competence	Civic behavior
School education	1	–	–	–	–	–
Civic literacy	0.382^**^	1	–	–	–	–
Civic cognition	0.258^**^	0.711^**^	1	–	–	–
Civic emotion	0.277^**^	0.777^**^	0.539^**^	1	–	–
Civic competence	0.332^**^	0.873^**^	0.494^**^	0.568^**^	1	–
Civic behavior	0.354^**^	0.871^**^	0.450^**^	0.510^**^	0.716^**^	1

**Correlation is significant at the 0.01 level (two-tailed).

### 4.4. Regression analysis

Apart from the statistical analysis above, the multiple regression analysis methods were also used to explore the relationship between senior primary school students’ civic literary and other independent variables and to test the account table rates and the predictive power that family SES and school education can do to the civic literacy of senior students. According to the research results, the multiple regression of family SES and school education on civic literacy scores makes the overall effect remarkable, *F*(2,1,294) = 146.418, *p*<0.001. As shown in the regression model summary (Table 13), the determination coefficient of the regression equation *R*^2^ = 0.185, that is, family SES and school education can explain 18.5% of the variance in civic literacy. According to Cohen’s rule of thumb, in the multiple regression analysis, the R^2^ values of the small, medium, and large effects are, respectively, 0.02, 0.13, and 0.26 (Chen, 2015); therefore, the coefficient of determination is moderate effect size. From the regression coefficients (Table 14), we could see it is obvious that both family SES [β = 0.190, *t* (1294) = 7.450, *p* = 0.000] and school education [β = 0.353, *t* (1294) = 13.825, *p* = 0.000] can easily predict senior students’ civic literacy scores. The effects of other variables are relatively small and, thus, were not included in the regression model. Therefore, the regression equation model of family SES and the school education to senior primary school students’ civic literary was constructed:


Y=262.937+1.238X1+2.515X2

**Table 13:** Family SES and school education regression model summary.

Model	*R*	*R* ^2^	Adjusted *R*^2^	SE of the estimate
1	0.430^a^	0.185	0.183	26.000

^a^Predictors: (constant), school education, family SES.

**Table 14:** Family SES and school education on civic literacy regression coefficients a.

Model		Unstandardized coefficients		Standardized coefficients	*T*	Sig.
		*B*	SE	Beta		
1	(Constant)	262.937	2.677		98.209	0.000
	Family SES	1.238	0.166	0.190	7.450	0.000
	School education	2.515	0.182	0.353	13.825	0.000

a. dependent variable: civic literacy.

Where: Y: criterion variable, representing the civic and literary scores of senior primary school students.

X1:predictive variable 1, representing family SES scores.

X2:predictive variable 2, representing the school education scores.

## 5. Conclusion, discussion and suggestions

Based on the measurement of civic literacy of senior primary school students and the study of its main affecting factors, the following conclusions were reached: The civic literacy of China’s senior primary school students is, on the whole, at a comparatively high level, and the scores on the four structural dimensions of civic literacy, from high to low, can be ranked as civic emotion, civic cognition, civic competence, civic behavior. Additionally, there is a significantly positive low correlation between family social and economic status (SES) and civic literacy and a significantly positive moderate correlation between school education and civic literacy. Moreover, family social and economic status (SES) and school education can, to a certain extent, interpret and predict the level of senior primary school students’ civic literacy but school education were better predictor than family social and economic status (SES). Further, the differences in civic literacy among senior primary school students are as follows: girls scored higher than boys; students who are or were student leaders scored significantly higher than those who were or were not. Civic literacy also varies by origin. Students in the city and the county scored significantly higher than those in the countryside areas. Students in the county also scored significantly higher than those in townships.

### 5.1. Educational compensation for civic literacy of senior primary school students in weak family social economic status

As for senior primary school students, family SES is their family background of growth. Social stratification is an important topic in sociology, and family SES is its core concept (Ji et al., 2020). Family SES refers to the family’s economic status and social status, both of which indicate a specific family and its members’ status in social stratification and whether it is strong or weak (Dong and Zhang, 2015). As some investigations and previous studies of the relationship between Family SES indicators and civic literacy showed, family SES significantly influences children’s awareness of rights and their emotions toward rights (Xu and Pang, 2020; Ren et al., 2021). As for children’s awareness of rights and their emotions towards rights, children in high family SES scored significantly higher than those in low family SES (Gong et al., 2012). Coherently, this study also indicated that family SES has a significantly positive low correlation with civic literacy of senior primary school students, and the former interprets and predicts the latter to a certain extent. Besides, senior students whose family is located in the city scored comparatively higher than the countryside, which corresponds to the overall level of civic literacy.

Pierre Bourdieu, the French sociologist, divides capital into social capital, economic capital, cultural capital and symbolic capital (Bourdieu, 1997). Generally speaking, if a family has a low SES, its economic capital and social capital are relatively weak, and correspondingly, the cultural capital and symbolic capital are relatively insufficient (Leonard et al., 2017; Ji et al., 2020). The civic literacy of senior students can be regarded as a kind of cultural capital (Keegan, 2021. As for students who grow up in a family of low SES, their cultural capital, including civic literacy, often tends to have “inherent disadvantages” (Pérez, 2021). The lack of civic literacy and cultural capital, which attach to students physically and mentally and the weakness of other capitals in these families, will have a combined effect on their development of civic literacy at school, making their civic literacy relatively weak. However, just as economic poverty is often not contributed by just one reason, the weak development of students’ civic literacy in a low-family SES is not entirely the result of the individual itself but largely a social result. Such vulnerable groups need help to get a fair status compared to others. “Compensating the disadvantaged” is a necessary idea for a civilized society, and ‘shaking off poverty on education’, from the point of view of the detail, should also include the educational compensation for the development of civic literacy of senior primary school students in a low family SES. Obtaining the appropriate educational compensation for developing civic literacy will be more likely to enable such a vulnerable group to contribute to the community and obtain well-being by themselves, thus helping to block the intergenerational transmission of a weak position. The specific educational compensation for civic literacy of senior primary school students with low family SES requires the society, especially the school to provide them with more civic education resources and implement effective civic education reasonably and pertinently.

### 5.2. Civic education for the specific condition of civic literacy of senior primary school students

Citizenship education is implemented through family education, social education, school education and self-education (Ma et al., 2021). The improvement of civic literacy of senior primary school students is highly related to family education and self-education (Mirzajonova and Parpiyeva, 2022). Meanwhile, the social environment also affects their civic literacy imperceptibly. Citizenship education is highly related to moral education (Halstead and Pike, 2006). Therefore, it faces the same dilemma as school moral education. That is, it lacks real attention and with low effectiveness in implementation. For the lack of real attention, civic education should own space be squeezed due to the objective existence of test-oriented education tendencies in school education. From the social perspective, civic education can help build a better society and can enhance the individual’s well-being from the individual point of view. As a person of great virtue is more likely to enjoy inner peace and well-being, good civic literacy will ultimately enhance the well-being of an individual. Based on this, facing the finding that there is still much room for enhancing civic literacy of China’s senior primary school students, it is the inherent requirement of primary school’s purport to leave space for civic education. Besides, on the practical level, it requires more and more social institutions to effectively implement civic education in primary school.

This study found that the scores on civic literacy of senior primary school students ranked from high to low: civic emotion, civic cognition, civic competence and civic behavior. As for individuals, civic emotion, like sympathy, is relatively easy to obtain. However, acquiring civic cognition and civic competence based on the former requires studying hard. Furthermore, it is more difficult for individuals to commit civic behaviors because it often needs to overcome more ‘fetters’. Therefore, the difference in the scores among the dimensions of civic literacy of senior primary school students that this study found is a realistic situation that can be easily interpreted theoretically. In addition, the study also found that the civic literacy of senior girl students is significantly better than boys girls’ civic literacy scores *M* = 299.53, SD = 28.15, *N* = 785 are significantly higher than the boys’ civic literacy scores *M* = 293.18, SD = 32.25, *N* = 857); the civic literacy of students who served or serving as student leaders is significantly better (*M* = 304.33, SD = 26.57, *N* = 649) than those who were not student leaders (*M* = 290.86, SD = 31.69, *N* = 984). As a whole, school education and civic literacy of senior primary school students are significantly positively correlated. Effective civic education at school can enhance the civic literacy of senior students, and the effectiveness of civic education needs to be based on the status quo of civic literacy found in this study. There should be a gender difference in civic education in the school. As a result, civic education should target different gender characteristics, especially different psychological characteristics. Civic education at school can also take measures like “serving as a class teacher, and schools should emphasis on females civic education and take more effort to reduce the discrepancy in this field between genders. It will enhance the civic literacy of senior primary school students. Moreover, school civic education needs to develop students” civic emotions, broaden their civic cognition, enhance their civic competence, and focus on promoting the implementation of their civic behavior. Such targeted initiatives will improve the effectiveness of civic education for senior primary school students, thereby contributing to the development of their civic literacy.

## Data availability statement

The original contributions presented in the study are included in the article/supplementary material, further inquiries can be directed to the corresponding authors.

## Ethics statement

The procedures performed in this study involving human participants were reviewed and approved by Zhejiang Normal University, Human Experiment Ethics Committee and were in accordance with the 1964 Helsinki Declaration and its later amendments and ethical standards. The patients/participants provided their written informed consent to participate in this study.

## Author contributions

YY, L-YC, and MO contributed to conception and design of the study. YY organized the database and performed the statistical analysis. YY and L-YC wrote the first draft of the manuscript. L-YC and MO wrote sections of the manuscript. All authors contributed to the manuscript revision, read, and approved the submitted version.

## Funding

This study was funded by research grant of Humanities and Social Sciences Project number 19YJA880023 from Ministry of Education, China.

## Conflict of interest

The authors declare that the research was conducted in the absence of any commercial or financial relationships that could be construed as a potential conflict of interest.

## Publisher’s note

All claims expressed in this article are solely those of the authors and do not necessarily represent those of their affiliated organizations, or those of the publisher, the editors and the reviewers. Any product that may be evaluated in this article, or claim that may be made by its manufacturer, is not guaranteed or endorsed by the publisher.
